# ADME Considerations and Bioanalytical Strategies for Pharmacokinetic Assessments of Antibody-Drug Conjugates

**DOI:** 10.3390/antib7040041

**Published:** 2018-11-30

**Authors:** Si Mou, Yue Huang, Anton I. Rosenbaum

**Affiliations:** Clinical Immunology and Bioanalysis, Clinical Pharmacology and DMPK, MedImmune LLC, 121 Oyster Point Boulevard, South San Francisco, CA 94080, USA; mous@MedImmune.com (S.M.); huangy@MedImmune.com (Y.H.)

**Keywords:** antibody-drug conjugates, ADC, bioanalysis, ADME, LBA, hybrid LBA-LC-MS/MS, PK

## Abstract

Antibody-drug conjugates (ADCs) are a unique class of biotherapeutics of inherent heterogeneity and correspondingly complex absorption, distribution, metabolism, and excretion (ADME) properties. Herein, we consider the contribution of various components of ADCs such as various classes of warheads, linkers, and conjugation strategies on ADME of ADCs. Understanding the metabolism and disposition of ADCs and interpreting exposure-efficacy and exposure-safety relationships of ADCs in the context of their various catabolites is critical for design and subsequent development of a clinically successful ADCs. Sophisticated bioanalytical assays are required for the assessments of intact ADC, total antibody, released warhead and relevant metabolites. Both ligand-binding assays (LBA) and hybrid LBA-liquid chromatography coupled with tandem mass spectrometry (LBA-LC-MS/MS) methods have been employed to assess pharmacokinetics (PK) of ADCs. Future advances in bioanalytical techniques will need to address the rising complexity of this biotherapeutic modality as more innovative conjugation strategies, antibody scaffolds and novel classes of warheads are employed for the next generation of ADCs. This review reflects our considerations on ADME of ADCs and provides a perspective on the current bioanalytical strategies for pharmacokinetic assessments of ADCs.

## 1. Introduction to Antibody-Drug Conjugates (ADC)

Antibody-drug conjugates (ADC) comprise a complex biotherapeutic modality composed of a warhead (cytotoxic drug) conjugated to a monoclonal antibody (mAb) via a chemical linker. ADCs are designed to selectively deliver the cytotoxic payload to tumor cells, while sparing normal tissues thus increasing the therapeutic index of anti-cancer therapy. To date, ADCs have been mostly used in oncology, primarily employing the IgG1 isotype scaffold [1]. While many ADCs are being tested in clinical trials, only a few have been approved for the treatment of cancer so far. The ADCs approved to date include trastuzumab emtansine (T-DM1, Kadcyla®, Genentech, South San Francisco, CA, USA), brentuximab vedotin (SGN-035, Adcetris®, Seattle Genetics, Bothell, WA, USA), inotuzumab ozogamicin (Besponsa™, Pfizer, New York City, NY, USA) and gemtuzumab ozogamicin (Mylotarg™, Pfizer, New York City, NY, USA). Trastuzumab emtansine targets Her2-positive metastatic breast cancer with a maytensine payload; the other approved ADCs are indicated for the treatment of hematological malignancies. Brentuximab vedotin is approved for the treatment of classical Hodgkin lymphoma, systemic anaplastic large cell lymphoma and primary cutaneous anaplastic large cell lymphoma by binding to the CD30 receptor and carries monomethyl auristatin E (MMAE) cytotoxic payload. Gemtuzumab ozogamicin is approved for the treatment of acute myeloid leukemia (AML) by targeting the CD33 receptor with a calicheamicin payload and inotuzumab ozogamicin is prescribed to treat relapsed or refractory B-cell precursor acute lymphoblastic leukemia (ALL) by targeting the CD22 receptor with a calicheamicin payload as well.

As mentioned above, ADCs inherently have a complex structure. Therefore, to evaluate their exposure-efficacy and exposure-safety relationships it is important to understand the absorption, disposition, metabolism and elimination (ADME) properties of the released warhead in addition to that of conjugated warhead and total antibody. These properties depend on the interplay of the antibody scaffold, conjugation site, linker and the conjugated warhead. Thus, in this review article we examine the ADME aspects of commonly used cytotoxic warheads, their conjugation strategies as well as bioanalytical assays employed to evaluate exposure of ADCs and their catabolites with a focus on mass spectrometry approaches.

### 1.1. Cytotoxic Warheads

**Figure 1:**
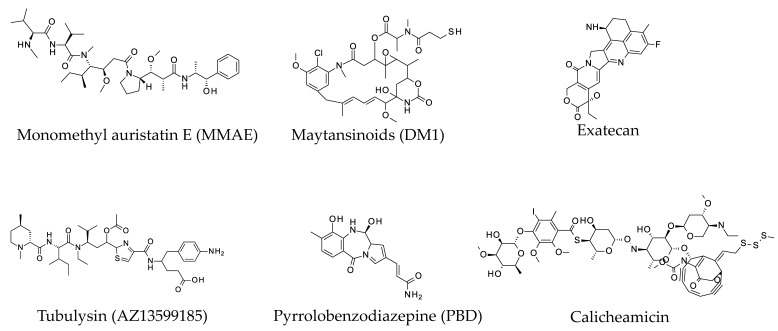
Structures of commonly used cytotoxic warheads.

Several different classes of warheads conjugated to antibodies are currently being developed for the treatment of cancer. The structures of some common warheads are displayed in Figure 1. One of the most clinically relevant classes of cytotoxic/cytostatic warheads consists of microtubule-disrupting agents. Auristatins, such as monomethyl auristatin E (MMAE) and monomethyl auristatin F (MMAF), are synthetic analogs of cytostatic dolastatins 10 and 15 [2]. These natural antimitotic drugs have been extracted from the sea hare, *Dolabella auricularia*. MMAE and MMAF auristatins emerged amongst various analogues due to combination of physicochemical properties, cytotoxic/cytostatic activity and in vivo stability. Additionally, availability of functional groups to enable conjugation to an amino acid side chain via either cleavable or non-cleavable linkers is required for candidate warheads [3]. Maytensine derivatives (DM) are another class of microtubule-disrupting agents used for conjugation to ADCs. Maytansine derivatives, such as DM1 and DM4, are analogues of a natural benzoansamacrolide product isolated from the bark of the African plant, *Maytenus ovatus* [4]. DMs disrupt microtubule polymerization by competing with the same site on tubulin as the anti-microtubule vinca alkaloids. As with auristatins, DMs have appropriate physicochemical properties and stability and can be readily conjugated to form ADC [5]. Tubulysins are antimitotic peptides originally isolated from myxobacteria and elicit their cytotoxic effects by inhibiting microtubule polymerization during mitosis [6]. AZ13599185 is a highly potent tubulysin warhead being developed by MedImmune/AstraZeneca (Gaithersburg, MD, USA) upon conjugation via four engineered cysteines to form MEDI4276, a biparatopic ADC against HER-2 [7,8]. MEDI4276 was evaluated in a Phase 1/2 Study in adult subjects with select HER2-expressing advanced solid tumors (NCT02576548) [9]. The second most commonly used ADC warhead class are DNA-damaging agents, such as pyrrolobenzodiazepines (PBD) [10,11,12,13,14], calicheamicins [15,16,17,18,19], duocarmycins [20] and novel topoisomerase inhibitors [21]. Unlike the warheads targeting microtubules which are cytotoxic for proliferating cells and cytostatic for non-proliferating cells, the DNA-damaging warheads are cytotoxic for both proliferating and nonproliferating cells. As with other cytotoxic warheads, PBDs have been derived from natural compounds. PBDs crosslink DNA in a site-specific manner by binding the minor groove the DNA helix. Several PBD analogues are currently being developed as ADCs [10,14,22,23]. Similar to PBDs, calicheamicin also binds the minor groove of DNA. However, instead of cross-linking DNA, calicheamicins cleave double-stranded DNA in a site-specific manner [24]. Recently, an ADC carrying a pyranoindolizinoquinoline topoisomerase I inhibitor, exatecan mesylate (DX-8951f), has shown promising clinical activity that appears to be distinct from trastuzumab emtansine [25,26].

### 1.2. Linkers and Conjugation Sites for ADCs

The conjugation of cytotoxic warheads to an antibody molecule is achieved via a chemical linker to the sidechain of an amino acid, such as the ɛ-amino group of lysines or the thiol residue of cysteines. The most frequently used linkers can be divided as non-cleavable or cleavable linkers (including enzymatically cleavable and chemically cleavable). For example, N-maleimidomethylcyclohexane-1-carboxylate is a non-cleavable linker and used in trastuzumab emtansine. Enzymatically cleavable linkers were applied in brentuximab vedotin (self-immolative para-aminobenzyl group linked via cathepsin-labile valine-citrulline dipeptide) and gemtuzumab ozogamicin (acid-labile hydrazone linker) has a chemical cleavable linker [27].

The selection of the conjugation site is critical for in vivo stability, metabolism and consequently pharmacological properties of an ADC. Through the development of antibody conjugation chemistry, there have been two main approaches to conjugate small molecule toxins through a linker to antibodies, resulting in a site specific or site non-specific conjugation. One approach is chemical conjugation of reactive amino acids within the protein scaffold. Most commonly used amino acids as conjugation sites for chemical conjugation are lysine and cysteine. However, other amino acids possessing reactive functional groups such as methionine [28], selenocysteine [29], and tyrosine [30] can also be used as conjugation sites. In some cases, non-natural amino acids can be added to the sequence to serve as specific conjugation site [31]. The second approach is to enzymatically conjugate the small molecule usually resulting in site-specific conjugation. In 2013, Zhou et al. employed glycoengineering to transform the N-glycosylation site for conjugation [32,33]. Later in 2014, Zhu et al. also reported using glycotransferase and chemically reactive sugar for a site-specific conjugation [34]. Sortase-mediated toxin conjugation was reported in 2017 [35]. Other enzymes utilized for antibody conjugation are coenzyme A analogs [36] and transglutaminase [37]. All approved ADCs to date employ the site non-specific, also known as stochastic approach for conjugation. Gemtuzumab ozogamicin, trastuzumab emtansine and inotuzumab ozogamicin are site-nonspecifically conjugated antibodies that all used lysine as the conjugation site. Brentuximab vedotin also employed the site non-specific conjugation to cysteine residues [38].

In the case of stochastic conjugation strategies, for a typical monoclonal antibody, there can be ~40–90 surface-exposed lysine and approximately 10 reactive residues [38,39]. Although the average drug-antibody ratio (DAR) can be reasonably well-controlled (usually around 3.5–4), the generated ADC drug substance may have a wide numerical distribution of DAR as well as many more positional isomers as the result of conjugation at different lysine sites. The non-specific conjugation created challenges for manufacturing, drug substance characterization as well as bioanalytical methods employed to determine ADC exposure. Therefore, there has been a continuous effort to better control the DAR distribution and improve the heterogeneity of the drug substance. The use of cysteine conjugation strategy instead of the one utilizing lysine has been demonstrated in recently approved brentuximab vedotin, where the cysteines used for interchain disulfide bonds were reduced and used for conjugation. This strategy, although resulting in a narrowed DAR distribution and reduced positional heterogeneity, is still considered as site non-specific, as there are eight possible sites for conjugation. In addition, using natural cysteine residues may lead to a disruption of the antibody structure. This may affect in vitro and in vivo the stability, pharmacokinetic half-life and efficacy of the ADC. The use of lysine and cysteine as conjugation sites has continued to develop over time. For cysteine conjugation, THIOMAB [40], cysteine re-bridging and cysteine insertion [41] have greatly improved the conjugation site-specificity with minimal interference to native disulfide bonds of the antibody. Although it is more difficult to achieve site specific conjugation for lysines, a method reported by Nanna et al. in 2017 demonstrated site-specific conjugation to the reactive lysine inside a hydrophobic pocket [39].

The selection of conjugation site affects the physicochemical properties of ADCs. Consequently, it may impact the stability, distribution and metabolism of the ADC in vivo. Additionally, the conjugation site selection can alter release kinetics of the warhead and further impact the efficacy, cytotoxicity and the therapeutic index. For instance, a study in 2008 by Junutula et al. indicated that site specific conjugation may be beneficial for increasing therapeutic index [42]. In 2013, Boylan et al. discussed the effect of conjugation site heterogeneity on the drug substance charge-state profile [43]. Scientists have noticed the conjugation site has an impact on the stability of the ADC [44,45,46]. Dorywalska et al. discussed the impact of the conjugation site on stability for both cleavable and non-cleavable linkers [46,47]. Furthermore, it has been demonstrated that the rate of metabolism will change according to the conjugation site [38].

Depending on the conjugation chemistry used, different drug/antibody ratios (DAR) can be achieved thus significantly modulating the therapeutic index of these compounds. The warhead, linker and mAb all contribute to the complexity of ADC’s overall absorption, distribution, metabolism/catabolism, and excretion (ADME) properties and ultimately affect clinical efficacy [48].

## 2. ADME Considerations for Pre-Clinical and Clinical Development of ADC

While the linker, warhead, and DAR can affect both in vitro and in vivo stability of the ADC as a whole, typically the biodistribution and PK of an ADC is dominated by the properties of the targeting antibody [49]. Unlike small molecule drugs, which are typically widely distributed, antibodies are restricted primarily to plasma and extracellular fluids compartments [50]. When a small molecule drug, such as a cytotoxic warhead is conjugated to a large molecule such as an antibody, its biodistribution is then primarily driven by the antibody component. This enables the toxin to selectively target tumor cells and tissues which would not be accessible by free form alone.

Initial catabolism of ADCs can be divided into two major aspects: Release of the free warhead from either intact or proteolytically degraded ADC and catabolism of the antibody component. The metabolism of the protein component can be affected by the scaffold employed as well as conjugation chemistry employed. Conjugation strategy can significantly affect the metabolism of the protein component if it employs residues that are important for overall stability of the protein scaffold.

Cytotoxic drug and its metabolites can be found in circulation after degradation of the ADC in target tumors, normal tissues and in circulation. As is the case for most small molecule drugs, free warheads are expected to partition into variety of tissues. The degree of distribution of the released warhead is the consequence of variety of processes that are determined by the toxin’s physicochemical properties, interactions with macromolecules (such as DNA binding in the case with DNA-damaging agents; and transformation of the drug in circulation as is the case of warhead release from maleimide-conjugates that may employ a retro-Michael reaction [51]) as well as being a substrate for transporters and pumps. Therefore, to fully appreciate the exposure-safety/efficacy relationships it is necessary to understand the mechanism of catabolism and metabolism of not only the conjugated drug but also its free form.

The distribution of an ADC in various tissues and subsequent deconjugation, catabolism and subsequent elimination influence ADC efficacy and safety. An ADC can be cleared from circulation by receptor-mediated endocytosis followed by degradation in the lysosomal compartment. Additional parameters that affect the pharmacokinetics of ADCs similarly to those of other antibodies include potential recycling via FcRn and clearance via the Fc-gamma receptor. Strategies that affect these parameters have been explored for some ADCs [7]. Furthermore, as with all biotherapeutics, ADCs can elicit anti-drug immune responses. Immune response can be raised against the protein portion of the molecule, the payload as well as the novel epitopes created by the conjugation [52,53,54]. Since ADCs have relatively narrow therapeutic margin, careful immunogenicity assessment is required for understanding of the potential impact of anti-drug antibodies on their PK/PD, safety and efficacy [55,56].

The mAb portion is mostly catabolized by proteolytic degradation [57]. In addition, an ADC is subject to nonspecific uptake (pinocytosis) and catabolism can happen in several organs such as liver, as is with traditional mAbs [58]. Moreover, DAR can also have a significant impact on ADC catabolism, in particular in cases of higher DAR molecules involving cysteine conjugation strategies [59,60]. The circulating unconjugated drug after ADC administration, on the contrary, has metabolic properties of small molecule compounds and has been reviewed extensively elsewhere [57,61,62,63].

Drug-drug interactions (DDIs) are an important consideration for small molecule drugs. However, typical systemic concentrations of the released toxin from an ADC are relatively low. Therefore, the risk of the ADC being a DDI perpetrator can be considered minimal [64]. On the other hand, due to the narrow therapeutic margin of the released warhead, the probability of a released drug to be a DDI victim should be carefully evaluated as the impact on patient safety can be high.

## 3. Bioanalytical Platforms for ADCs

In order to capture the PK profiles of ADC as well as their major catabolites, complementary bioanalytical methods must be considered that can interrogate both protein and small molecule components of these drugs. In the case of released warhead assays, traditional small molecule approaches of employing liquid chromatography coupled with tandem mass spectrometry (LC-MS/MS) [65] or enzyme-linked immunosorbent assay (ELISA) [66] can be applied. Commonly used bioanalysis platforms to examine exposure–response relationships for biotherapeutics include ligand-binding assays (LBA) and hybrid LBA-LC-MS assays [67,68]. These assays have been developed and extensively employed to perform quantitative analysis of ADCs in biological matrices. Table 1 summarizes the advantages and challenges of the immunoassay (LBA) platform and hybrid LBA-LC-MS platform.

In 1971, ELISA was introduced to measure analyte concentrations which employed microplate-immobilized antigens and enzyme-linked anti-immunoglobin antibodies [69]. ELISA is currently one of the most commonly used LBA for the quantification of diverse analytes. Recent advances in signal amplification and pre-enrichment of low-abundance analytes in biological matrices enabled highly sensitive LBA methods for the quantitative analysis of variety of analytes [70,71,72,73]. For the bioanalysis of ADCs, LBA has been routinely used for the measurements of total antibody as well as some conjugated warhead assays. Most generic or analyte-specific LBA methods benefit from straightforward design and operation. While providing adequate measurements of total antibody, LBA methods for ADC bioanalysis are typically not sensitive to the measurement of the drug-to-antibody ratios (DAR) or the overall drug load [67,68,74]. Selectivity and specificity of the LBA assays is defined by the capture and detection of antibodies. Thus, possible changes during reagent manufacturing, as well as any subsequent derivatization (such as biotinylation or conjugation with fluorochromes), can potentially affect their selectivity and/or binding efficiency [75,76,77]. Consequently, careful bridging and/or qualification of these critical reagents from lot to lot is necessary.

Hybrid LBA-LC-MS platform combines the immuno-affinity enrichment of analyte with separation of complex mixtures using liquid chromatography and detection of analyte(s) using mass spectrometry. By separating highly complicated mixture using liquid chromatography and by measuring known and specific mass-to-charge ratio of an analyte using mass spectrometry, LC-MS or LC-MS/MS can enable highly selective and specific identification as well as accurate and precise quantification for bioanalysis of ADCs. Since LC-MS is able to measure both small and large molecules, this enables analysis of complex biotherapeutics that require multiple assays including antibody-conjugated drug, total antibody and intact ADCs [60,78,79,80,81]. By employing tandem mass spectrometry, LC-MS/MS can identify specific proteins by directly sequencing surrogate peptides as well as detailed structural information beyond sequence such as post-translational modifications, macromolecular complexes characterization and analysis of DAR distribution and dynamics [60,82,83,84,85].

For the detection of trace-level released toxin species from ADCs in the circulation, competition ELISA has been commonly developed and used. Studies have reported to quantify released MMAE [86], maytansinoid [74,87,88] and calicheamicin [66,89] utilizing ELISA-based methods. Alternatively, LC-MS methods can be used to quantify free drug catabolite [65]. This usually involves traditional small-molecule sample preparation steps for LC-MS analysis which includes protein precipitation and/or solid-phase extraction (SPE) to remove the plasma proteins prior to LC–MS/MS.

As mentioned above, anti-drug antibodies (ADA) can impact biotherapeutic efficacy, PK and safety. Moreover, ADA can impact the quantification of biotherapeutics by hybrid LBA-LC-MS/MS methods by interfering with either the capture or digestion efficiency of the analyte. However, since it is not possible to know a priori what epitopes would be susceptible to ADA or what the affinity and concentrations of ADA would be, it is not possible to evaluate the potential impact of ADA on bioanalytical method performance during early stages of ADC assay development.

## 4. Hybrid LBA-LC-MS for ADC Analysis

**Figure 2:**
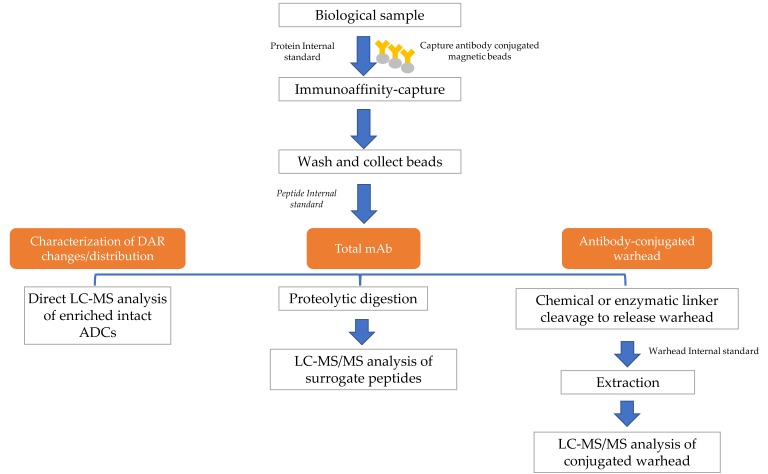
A typical workflow for bioanalysis of antibody-drug conjugates (ADCs) using hybrid LBA-LC-MS (ligand binding assay - liquid chromatography coupled with mass spectrometry).

A typical hybrid LBA-LC-MS assay includes immuno-capture of the analyte, followed by the enzymatic digestion for total antibody analysis or cleavage of drug linker for antibody-conjugated drug analysis. Alternatively, the captured analyte can be eluted without digestion for direct measurement of intact ADCs for DAR distribution and additional biotransformation characterization (Figure 2).

### 4.1. Hybrid LBA-LC-MS of Surrogate Peptides of ADCs

The surrogate analyte (as known as surrogate peptide) method employing immuno-capture and proteolytic digestion is commonly used to quantify total antibody and determine drug levels for PK assessments [90,91,92]. Similar to traditional LBA, the capture reagents vary based on their specificity from such generic capture reagents as protein A, protein G to species-specific IgG, which can be additionally isotype specific. More specific capture reagents include target antigens and anti-idiotype (anti-ID) antibodies. Moreover, for certain payloads, anti-payload antibodies can be employed [93,94]. Direct detection of the total antibody using surrogate peptides typically results in assays with good linear response, wide dynamic range and great sensitivity, with limits of quantification in the range of low ng/mL or even pg/mL of plasma/serum ADC concentration [78,93].

Selection of suitable surrogate peptides from either light chain or heavy chain with good sensitivity and high specificity is critical to the quantitative accuracy and reliability of the assay. It is important to carefully evaluate both immuno-capture and proteolytic digestion efficiencies in order to develop a robust and sensitive method [95,96]. Stable isotopically labeled internal standards (SIL-IS) are usually incorporated as early in the workflow as possible to normalize as much of the process as possible for improved accuracy and precision of quantification. However, when adding SIL-IS before immuno-capture, competitive binding of the protein internal standards to the capture reagent needs to be carefully evaluated as it may affect quantification accuracy. Thus, typically, capture reagents are used in significant excess of the analyte and experiments evaluating capture efficiency must be conducted, in particular at the concentrations at the upper limit of quantification to ensure adequate capture efficiency throughout the quantification range.

For highly specific MS detection, triple quadrupole (QQQ) mass spectrometers are commonly used to target the surrogate peptides of interest. Surrogate peptides are usually fragmented using QQQ to improve selectivity, specificity and dynamic range of the assay. Although LC-MS technology is quite mature with automated injection and sample analysis, instrument time per sample remains longer compared to ligand-binding methods. Additionallt, the multiple steps of semi-manual sample preparation from immuno-capture to proteolytic digestion remain laborious which results in lower throughput of analysis especially for large-scale pharmacokinetic studies. To increase sample preparation efficiency, automation of the hybrid LBA sample preparation platform have been increasingly employed and applied for immuno-capture and protein digestion [97,98,99]. With the automated sample preparation platform, it is critical to optimize the pipetting parameters for magnetic bead-related steps such as consistent aspiration of bead slurry and minimal loss of beads during wash steps. In recent years, a few generic hybrid LBA LC-MS workflows have also been commercialized and accessible for bioanalytical researchers. Some of these commercially available workflows incorporate reagents, optimized protocols, automation and LC-MS methods, saving time for method development.

### 4.2. Hybrid LBA-LC-MS of Intact ADCs

Direct measurement and characterization of intact ADCs after immuno-enrichment has been an evolving research topic as LC-ESI-MS technology has been improving in recent years. The 2002 Nobel prize was awarded to John Fenn and Koichi Tanaka for their work on developing soft ionization methods for mass spectrometry which significantly expanded the application field of MS to sophisticated intact biological macromolecules [100,101]. Nowadays, state-of-the art orbitrap technology drives high-resolution and accurate mass (HRAM) data, providing measurements of over 100 k resolution at 1000 m/z. Accurate mass assignment of orbitrap can be achieved with sub-1-ppm mass accuracy of analytes. Time of flight (TOF) instrument is another HRAM mass analyzer which can achieve analysis of intact biomolecules at mass resolution of over 50 k resolution at 1000 m/z and mass accuracy below 10 ppm [102]. While orbitrap instruments provide high sensitivity and resolution, TOF instruments are capable of fast full scan data acquisition for quantification of biomolecules. Significant improvements in different mass spectrometry technologies enable reliable and robust qualitative and quantitative analysis of intact ADCs in a single analysis [103].

Compared with more traditional biotherapeutic modalities such as monoclonal antibodies, therapeutic proteins and peptides, the heterogeneity of the ADCs resulting from combinatorial complexities of the antibody scaffold, linker and conjugated warhead contributes to a significantly increased structural complexity [104]. Therefore, further sophistication of bioanalytical methods employed to interrogate the exposure-efficacy and exposure-safety relationships is required. A better understanding of DAR distribution, fraction of unconjugated mAb in vitro and in vivo is needed especially for PK/PD studies [105]. A few novel methods with hybrid immuno-affinity LC-MS have demonstrated direct quantification of released toxin by quantifying DAR distributions of the ADC in circulation using high resolution accurate mass spectrometry (HRAM-MS) [60,81,82,84,85,106]. While this intact LC-MS analysis approach provides a new aspect of direct measurement and structural characterization of ADCs, there still remain challenges related to assay sensitivity and dynamic range, obtaining internal standards for intact ADCs, relatively poor chromatographic peak shapes and limited separation resolution of intact molecules with different DAR values and additional complexity from potential biotransformations. In order to overcome such bioanalytical challenges, sophisticated chromatographic and/or HRAM methods need to be employed.

### 4.3. Hybrid LBA-LC-MS of Conjugated Drugs

Using LC-MS/MS to quantify conjugated drug concentration provides direct information on drug load and is highly sensitive to any changes in the drug load. After isolating the ADC from a matrix of interest using immuno-capture, the linker of ADC is cleaved by chemical reagents or enzymes depending on the linker structure followed by LC-MS/MS analysis of the released drug [65].

## 5. Discussion

Antibody-drug conjugates are a unique class of anti-cancer biotherapeutic agents that have recently demonstrated appreciable clinical utility. Due to the inherent heterogeneity of this class of compounds their ADME properties are correspondingly complex. This complexity requires sophisticated bioanalytical assays for the assessments of not only intact ADC but also that of total antibody and released warhead to properly assess their catabolism, subsequent distribution and elimination. Both LBA and hybrid LBA-LC/MS methods have been employed to assess PK of ADC and total antibody, while competitive ELISAs and traditional LC-MS/MS have been employed for free warhead detection assays. Future advances in bioanalytical techniques, particularly in mass spectrometry applications to the field of intact protein analysis would be needed support more comprehensive analysis of in vivo biotransformation and DAR distribution of ADCs. This has become more relevant as more innovative conjugation strategies, antibody scaffolds and novel classes of warheads are being employed for the next generation of ADC candidates. The resulting additional degree of understanding of the analytical complexity of ADCs’ metabolism and disposition should begin to inform our interpretation of exposure-efficacy/safety relationships as well as the hitherto largely unexplored interplay of biotherapeutics metabolism and anti-drug immune responses. Hence, a more thorough understanding of these parameters is critical for the design and subsequent development of future clinically successful ADC therapeutics.

## Figures and Tables

**Table 1:** The advantages and challenges of the ELISA-based immunoassay platform and hybrid ligand binding LC-MS (liquid chromatography-mass spectrometry) platform.

Assay	Advantage	Challenges
LBA	Sensitive quantitative analysis for large molecules	DAR insensitive; does not provide measurement of the DAR or the overall drug load
	Low equipment cost	Typically not sensitive to biotransformation
	High throughput	Specificity and selectivity is determined only by the capture and detection antibodies
	Easy to implement	Lack of structural/sequence information of the ADCs
		Potential cross-reactivity between antibodies in a multiplexed immunoassay
		Limited multiplexing capability
		Time-consuming to develop highly selective and specific antibodies
Hybrid LBA-LC-MS	Sensitive quantitative analysis for complex biotherapeutics such as ADCs	Relatively higher equipment cost compared to LBA assays
	DAR sensitive--able to measure DAR/drug load	Complexity of instrument operation and data interpretation
	Specificity and selectivity achieved using antibody capture, chromatographic separation and characteristic fragmentation of surrogate peptides	Lower throughput due to additional steps such as proteolytic digestion and chromatographic separation requiring samples to be injected one at a time
	Able to provide ADC analyte structure information	Relatively low sensitivity for intact ADC analysis
	Can be sensitive to biotransformation	Reliance on surrogate analytes for quantification
	Could be highly multiplexed; many analytes can be analyzed at a time in a single LC-MS analysis	

LBA, ligand-binding assays; DAR, drug-antibody ratio; ADCs, Antibody-drug conjugates; LBA-LC-MS, LBA-liquid chromatography coupled with mass spectrometry.
